# Increasing the efficiency of the herd reproduction system by introducing innovative technologies into dairy farming in Northern Kazakhstan

**DOI:** 10.14202/vetworld.2021.3028-3037

**Published:** 2021-11-28

**Authors:** Vitaly Anatolevich Raketsky, Askar Myrzakhmetovich Nametov, Vasily Arkadyevich Sozinov, Abdrakhman Abdybekuly Baisakalov

**Affiliations:** 1 Department of Veterinary Medicine, NAO Kostanay Regional University Named after A. Baitursynov, Kostanay, Republic of Kazakhstan; 2 West Kazakhstan Agrarian and Technical University Named after Zhangir Khan, Uralsk, Republic of Kazakhstan; 3 Department of Therapy, Surgery, Obstetrics, and Infectious Diseases, Vyatka State Agrarian and Technological University, Kirov, Russian Federation; 4 Laboratory of Livestock Breeding, SHOS Zarechnoye LLP, Kostanay, Republic of Kazakhstan

**Keywords:** AlphaVision visual insemination system, biotechnological reproduction methods, cervicitis, diseases of the reproductive organs of cows, fertility rate, sexed semen

## Abstract

**Background and Aim:**

In recent years, Kazakhstan has increasingly imported breeding cows for dairy and beef production. To maintain and improve their breeding qualities of reproductive function, it is necessary to constantly monitor the herd reproduction system. The aim of this study was to increase the level of herd reproduction by introducing innovative technologies into dairy farms in Northern Kazakhstan. To achieve this goal, the AlphaVision visual insemination system (IMV Technologies, France) was used, aiding to improve the artificial insemination method in farms in Northern Kazakhstan and increased the breeding rate using sexed semen to inseminate cows. In addition, the AlphaVision device was used in the differential diagnosis of certain diseases of the reproductive organs of cows.

**Materials and Methods:**

The object of the study was 200 cows (3-5-year-old) and 100 heifers (16-18-month-old) of Holstein breed. The authors carried out a comparative analysis of biotechnological methods of reproduction – the cervical method insemination with rectal fixation of the cervix (traditional method of insemination) and the AlphaVision visual insemination system, and the effectiveness of AlphaVision for diagnosing some reproductive tract abnormalities in cows was studied. In the experiment on conducting artificial insemination through AlphaVision, we have used both normal (two-sex) and sexed semen.

**Results:**

When using the AlphaVision visual insemination system, a higher percentage of fruitful insemination was noted (20.7%) than when using the traditional method. The images obtained with AlphaVision made it possible to identify cows with abnormal sexual cycles, signs of vaginitis, endometritis, cervicitis, and differentiate them by the nature of the exudate. In many cases, visual examinations of the vagina and cervix are not carried out before the traditional method of artificial insemination. For this reason, some vaginal and cervical abnormalities are not diagnosed, resulting in reduced fertility in cows. We have found that the number of genital abnormalities has increased by 30% with the increasing age of cows. Obstetric and gynecologic pathologies in high-yielding cows are noted in more than 50% of the herd. A comparative assessment of clinical manifestations of cervicitis and other pathologies of reproductive organs, using the AlphaVision visual insemination system, has been carried out for the identified diseases. With the traditional method of insemination with conventional semen, the calf yield per 100 cows for the period 2016-2019 has been 65-80% and with sexed semen 30-50%. With AlphaVision in 2020, the insemination rate was 85% conventional and 60% sexed, respectively, which was 5% and 10% higher than with conventional insemination. This was due to the improved diagnosis of some reproductive diseases in cows.

**Conclusion:**

The introduction of innovative technology, namely, the visual insemination system AlphaVision, into the practice of dairy farms in Northern Kazakhstan increased the level of the herd reproduction system.

## Introduction

To reach the genetically determined level of milk productivity of cattle, it is necessary to maintain a high level of herd reproduction, ensure timely fruitful insemination of cows and heifers, and annually receive viable offspring from them [1]. A huge role here is played by biotechnological methods of reproduction, both from the point of view of increasing the efficiency of breeding work and increasing the reproduction of the herd. To solve this problem, it is necessary to intensify the reproduction of animals [2].

The world’s population is growing every year. To meet the increased demand for food protein by 2050, it will require a two-fold increase in animal genetics to meet the increased [3,4]. An important condition for solving this task is to increase the reproductive function of animals and poultry. The vast majority of livestock and poultry are currently produced by artificial insemination, including 70% of cattle, 90% of pigs, and 100% of turkeys, except for some traditional breeds [5]. Embryo transplantation is expedient when setting goals for accelerated qualitative renewal of the herd with a radical increase in productivity over the next 2-3 years. It allows increasing the genetic potential of the breeding nucleus in dairy and beef cattle breeding 5 times faster than with artificial insemination. Besides, one donor with a high-value genetic potential can give the farm ten or more calves annually [6-8]. At the moment, in the Kostanay region, the issues of embryo transplantation into cattle remain open.

All of the above largely depends on the reproductive health of the broodstock of dairy cattle. In modern dairy farming, an urgent problem is pathological condition of the reproductive organs of cows after labor, as well as their differential diagnosis and treatment [9-11]. For example, when at least one obstetric pathology is diagnosed in the postpartum period, experience a decrease in fertility with the first insemination [12-14]. At the same time, there are no specific diagnostic criteria to differentiate functional disorders from inflammatory diseases of the uterus. Therefore, one of the important conditions for the development of cattle breeding is the improvement of existing methods, as well as the search for new methods for diagnosing diseases of the reproductive organs in cows [15,16].

In dairy farming, one of the most time-consuming processes is the reproduction of cattle. The milk productivity of cows, the efficiency of selection and breeding work, the duration and intensity of the use of genetically valuable highly productive animals, the economy, and profitability of production depend on the level of reproduction of the herd. The relatively short period of intensive production use of dairy cows requires the annual introduction of 25-30% or more highly productive first calving cows into the main herd. It becomes impossible with a significant decrease in the level of reproduction, calf crop, and their weak preservation [17,18]. In solving this problem, the replenishment of herd method using sexed semen stands out. In this way, it is possible to increase the number of animals faster than with traditional methods and to replace discarded cows in a dairy herd with greater efficiency. The use of sexed semen in animal husbandry makes it possible to obtain over 80% of heifers from all received calves. This, in turn, allows renewing the dairy herd with first-calf heifers in a shorter time [19]. However, researchers and practitioners have no consensus on the use of sexed semen [20,21]. Some literature data show that, with obvious advantages, this method reduces the percentage of cows’ pregnancy. Therefore, questions on the study of the effect of sexed semen on the reproductive qualities of animals are relevant as well.

Improvement of biotechnological methods of reproduction using methods of the replenishment of herd with young high-yielding animals inseminated with sexed semen, diagnosis of pathology of the genitals in cows with the use of new technologies and innovative solutions leads to an increase in the efficiency of the herd reproduction system. Thus, the issues under consideration in the reproduction of the herd in North Kazakhstan are extremely relevant.

The study aimed to review the innovative technologies in the reproduction of dairy cattle breeding in Northern Kazakhstan. The objectives of the study included a comparative analysis of biotechnological methods of reproduction [the cervical insemination with rectal fixation of the cervix using the AlphaVision visual insemination system (IMV Technologies, France)], studying the use of AlphaVision in the diagnosis of some diseases of the reproductive organs of cows, and the replenishment of the herd with young cows obtained by insemination with sexed semen.

## Materials and Methods

### Ethical approval

The research protocol was discussed and approved at a meeting of the local ethical committee of the A. Baitursynov KRU non-profit joint-stock company belonging to the Ministry of Education and Science of the Republic of Kazakhstan, dated February 3, 2019.

### Study period and location

The research to study the effectiveness of the herd reproduction system by introducing innovative technologies in dairy cattle breeding in Northern Kazakhstan was carried out based on the V. Dvurechensky Agricultural Institute, A. Baitursynov Kostanay Regional University (KRU), the experimental part was carried out at the Olzha-Ak-Kuduk LLP of the Kostanay district and Turar LLP of the Fedorovsky district, Kazakhstan. Collection and analysis of documentation and statistical data from farms were carried out from March 2016 to May 2020. The experimental part of the study was carried out in May 2019 and March-May 2020.

### Studies 1 and 2

A comparative analysis of biotechnological methods of reproduction and determining the most rational and effective method inseminating cows, as well as the effectiveness of the AlphaVision device for diagnosing some reproductive organ abnormalities in cows, were carried out in Olzha Ak-Kuduk LLP, Kazakhstan. The objects of research were 200 cows of 3-5 years of age of Holstein breed, black and white color. The live weight of the cows was 650-750 kg.

The cows were kept in loose housing; feeding was carried out with a common mixed feed, balanced per their physiological needs. The cows were milked two times a day in the morning and in the evening; milk yield was recorded at each milking. Milk was analyzed for fat, protein, and somatic cells in a total milk sample from experimental and control cows. The experimental and control cows had no signs of ketosis, acidosis, lameness, and displaced abomasum. Before insemination, all cows were checked for estral mucus.

**Figure-1:**
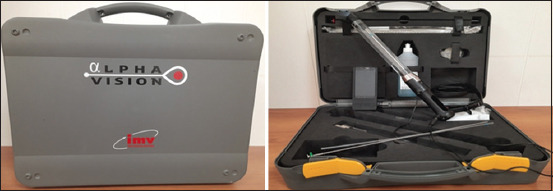
AlphaVision visual insemination system (IMV Technologies, France).

In May 2019, in Olzha Ak-Kuduk LLP, Kazakhstan, we conducted a study to determine the effectiveness of different insemination methods. The cows were divided into two groups, the control group, and the experimental group, with 50 cows in each. Cows were of the same age and weight with no problems with insemination. In the control group, insemination was performed with the traditional method, and in the experimental group, it was done with the AlphaVision visual insemination system (IMV Technologies) (Figure-1). Results were determined at the onset of pregnancy in inseminated cows.

In March-April 2020, the effectiveness of the AlphaVision device was studied for the diagnosis of some pathology of the reproductive organs of cows. The cows were divided into two groups for the experiment: 50 cows in the control group (the 3^rd^-year-old ones had problems with insemination) and 50 cows in the experimental group (the 5^th^-year-old ones had not inseminated either).

Work with AlphaVision was carried out according to the instructions as follows. We started by putting on the neck extension for the video terminal, and then inserted the video terminal into the case through the hole provided for this. Then the video terminal was connected to the Micro-USB connector. Then the clip of the neck extension was opened to insert the video terminal there, after which the other end of the cable was connected to the AlphaVision device so that the two red dots were opposite each other. After that, the terminal was turned on. The charge needed to be at least 50%. Then, we launched the application. In the application, we hovered the cursor over OK and clicked on it. It was also necessary to make sure that the image was of good quality. Next, we installed the mirror on the AlphaVision pistol and put the cable around the neck. After that, a special sanitary shirt was put on the equipment.

Before the study was carried out, the condition of the cow’s vagina and cervix was checked, the external genitalia of the cow were cleaned with a warm 0.02% solution of furacilin. B-LUBE gel was laid on a sanitary shirt and around the mirror. Opening the vaginal slit, the camera was inserted into the vaginal vestibule at an angle of 45 degrees. Then, aligning it parallel to the rectum, it was inserted further into the vagina. Next, we advanced the AlphaVision pistol to the cervix and observed using the image transmitted to the terminal. Thus, we had started to check the condition of the cow’s vagina, cervix using the resulting image on the screen.

### Assembly of a Kombicolor insemination gun (IMV Technologies)

We performed the process as directed and started by holding the Kombicolor syringe in one hand, then pulling back the plunger of the Kombicolor syringe and pushing it into the graduated extension. After that, the Kombicolor colored ring was fixed to the end of the graduated extension, then the steel ring of the Kombicolor syringe plunger was moved into the provided groove on the pusher so that it snapped into it. The pusher was then inserted into the graduated extension at the first mark.

The preparation of the sperm straw was started by defrosting and drying. After that, we cut off the sealed part of the straw with the cutter supplied. The straw was inserted into the chamber of the Kombicolor syringe and the Alpha tubing was then inserted into the Kombicolor syringe until completely blocked. Next, the pistol was inserted into the AlphaVision device at the first mark. After that, we pumped the Kombicolor syringe.

### Insemination

Before artificial insemination, the external genitalia of the cows were cleaned, followed the same steps as in conventional insemination. First, the B-LUBE gel (IMV Technologies) was applied around the speculum at a distance of 10 cm from its edge and the edge of the speculum was brought closer to the vulva. Then, the vulva was moved apart, opened the vaginal slit, and inserted the mirror into the vagina from below at an angle of 45 degrees. Then, after passing the vaginal fornix, the speculum was aligned in the direction of the vagina. Then, the AlphaVision apparatus was advanced to the cervix using the image transmitted to the terminal. After inserting a Kombicolor insemination syringe into AlphaVision, the graduated extension was pushed in until the insemination tube appeared on the image at the entrance to the cervix. One mark on the handle corresponds to 1 cm. The Alpha insemination tube was then positioned opposite the entrance of the cervix and inserted into the uterus while continuing to push the gradual extension (rectal palpation was used to make sure that the syringe was positioned correctly). After that, a dose of sperm was injected by gently pressing on the pusher until it stopped, trying not to move the graduated extension. We checked the cervix in the image, making sure there was no outflow of semen. After emptying the straws, the AlphaVision extracted back.

In the control group, when diagnosing diseases of the reproductive organs, the methods of rectal and vaginal examination were used. In the experiment with insemination cervical method with rectal fixation of the cervix, the following instruments were used: An ampoule, a plastic catheter 35-40 cm long, and an obstetric polythene glove. During the experiment, the air was removed from the ampoule; the ampoule was filled with prepared sperm and connected to the catheter. Performed cleaning of the external genitalia of the heifers; a glove was put on the hand and lubed with gel. Thus, a prepared hand was inserted into the rectum, through the wall of the cervix was groped and clasped and fixed with two fingers (the second and third), and with the thumb, we felt the cervical opening, where the catheter was inserted. On the other hand, the animal’s genital slit was opened, and the catheter was inserted into the vagina and then into the cervical canal and the sperm was injected. Then, we pulled out the hand, and then the catheter. At the same time, the clitoris was massaged through the vulva within 2-3 min.

We used sexed sperm to produce single-sex calves purchased fromTaurus LLP, Almaty, Kazakhstan. The semen was produced in the USA from three stud bulls of Holstein breed MARVEL №551HO03444, CORSAIR №151HO03128, and REDROK №551HO03501 in 2015. The planned milk productivity of the broodstock, on which the experimental work was carried out, is 8000 kg of milk per year, with a milk fat content of 3.75%. All experimental animals were kept in the same feeding and housing under operating conditions.

### Study 3

Scientific and industrial research to study the effectiveness of using AlphaVision in insemination with sexed semen was carried out in Turar LLP, Fedorovskiy district of Kostanay region, Kazakhstan, in March-May 2020. On the farm, artificial insemination was performed by the cervical method with rectal fixation of the cervix. Farms, where studies were conducted, were free from infectious and invasive diseases. Collection and study of documentation (veterinary and zootechnical registers) were carried out from 2016 to 2020. The object of the research was heifers of mating age of the Holstein breed, 16-18 months of age, weighing 390-410 kg, and black-and-white color. The heifers were kept by untethered method and fed a common mixed diet twice a day, balanced according to their physiological needs. Heifers were kept separately in stalls equipped with tunnel ventilation and sand-bed stalls. During the experiment in March-May 2020, the animals were divided into two groups: the control group and the experimental group, 50 heads in each. Heifers of mating age included in the control and experimental groups were clinically healthy. In the control group, insemination was carried out with non-divided sperm, and in the experimental group with sexed semen. The animals were inseminated during the heat, which was detected with a Draminski electronic detector (Draminski, Poland), an estrometer and recording of animal movement activity. If the heat was revealed in the morning, the procedure on insemination was carried out in the evening at 17-19 h. If heat was observed in the evening, then the insemination procedure was carried out the next morning, at 5.00-6.00 O’clock. Artificial insemination was performed using AlphaVision, once. Hormonal preparations for synchronizing estrus of cows were not used during sex-segregated semen use. During the experiment, all animals were in the same conditions of feeding and housing.

### Statistical analysis

The research results were processed by the method of variation statistics with the Microsoft Office software package using the Excel software (Microsoft Office, USA).

## Results

### Experiment 1

In the course of the experiment in the comparative analysis of biotechnological methods of reproduction (the traditional method and the method with the AlphaVision visual insemination system), it was found that among the 50 animals inseminated by the traditional method, 48 heads had been inseminated – two animals were unsuitable for insemination due to abnormalities in the genitalia. In this group, 1 month after insemination, 28 animals (58.3%) were found to be pregnant. In a test group, of 50 animals, 43 heads were inseminated by the AlphaVision visual insemination system, of which 1 month after insemination, when checked for pregnancy by ultrasound, 34 cows were pregnant (79,0%). The remaining seven heads in the experimental group and two in the control group were sent to treatment. A detailed data set of the study results is found in Table-1.

**Table-1:** Comparative efficiency of cow insemination methods.

Indicator	Groups	

	Control group	Experimental group
All cows	50	50
Of those, cows with signs of heat	50	50
Visualization of pathology before insemination	2	7
Inseminated	48	43
Insemination time 1 head	Up to 5 min	Up to 4 min
Pregnant	28	34
Price of 1 dose of semen, tenge	1200	1200
Cost of all doses, tenge	115,200	103,200
Percentage of fruitful insemination, %	58.3	79.0
Insemination index	1.71	1.26

In determining the most effective method of insemination, the time required for insemination, the cost of sperm, the cost of sperm, and the fertilization rate were considered. Two cows in the control group showed purulent-catarrhal endometritis on further examination of estral mucus. The visual insemination system revealed genital abnormalities with signs of endometritis and cervicitis in seven cows, which is 10% more than in the control group.

**Figure-2:**
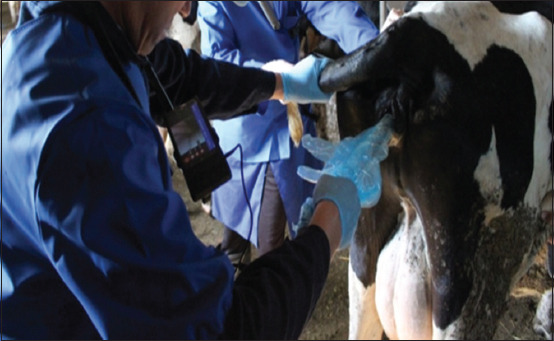
Artificial insemination using the AlphaVision visual insemination system (IMV Technologies, France).

**Figure-3:**
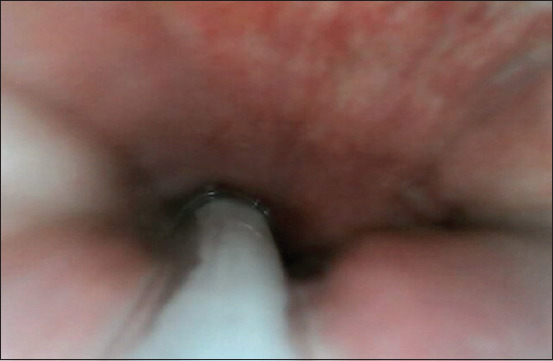
Catheter insertion into the cervical canal with the AlphaVision visual insemination system.

The table shows that the use of the AlphaVision visual insemination system at Olzha Ak-Kuduk LLP, Kazakhstan made it possible to identify more cows with signs of the estrous cycle with abnormalities in the endometrium, while in the control group, 4% were identified, and in the experimental 14%. A higher percentage of fruitful insemination was established by 21% and in the experimental group (Figures-2 and 3). It should be noted that the insemination assistance system, equipped with a sealed chamber, provided a significant increase in the convenience of the inseminator. In particular, it is possible to check the involution of the cow’s cervix, showed whether there was any pathology, and simplified the determination of its location for more painless insemination.

### Experiment 2

One of the causes of infertility in cows is genital abnormalities, which are widespread in large dairy complexes. For example, we found that under conditions of Olzha Ak-Kuduk LLP, Kazakhstan, obstetric and gynecological pathologies in highly productive cows were reported in more than 50% of the livestock. Meanwhile, subinvolution of the uterus was observed in 16%, cervicitis was diagnosed in 11.3%, and vaginitis in 8% of infertile animals. The spread of diseases reproductive organs of cows depending on age is shown in Table-2.

**Table-2:** The spread of pathology of the reproductive organs depending on age, diagnosed with visual insemination system AlphaVision.

Indicator	AlphaVision Visual Insemination System in the use of diagnosis of certain reproductive organ diseases			

	Control group: 3 years		Experimental group: 5 years	
	
	Heads	%	Heads	%
All cows	50	100	50	100
Of those, cows with signs of endometritis	15	30	18	36
Cervicitis	2	4	5	10
Vaginitis	2	4	4	8
Cervicalerosion	1	2	2	4
Cervicalcysts	-		1	2
Cervicalpolyps	-		3	6
Myometritis	-		1	2
Subinvolution of the uterus	6	12	7	14
Total	26	52	41	82

Analyzing the data in Table-2, we can note that with an increase of age of the cows, the incidence of postpartum pathologies also increased. For example, in Olzha Ak-Kuduk LLP, Kazakhstan, the number of genital abnormalities increased by 30% with the increasing age of cows. For the identified diseases, a comparative assessment of the clinical manifestations of cervicitis and other pathologies of the reproductive organs (Table-3) was carried out using the AlphaVision visual insemination system and rectal palpation. The clinical picture of obstetric and gynecological abnormalities in cows has been studied (Table-4).

**Table-3:** Manifestation of clinical signs of certain genital pathologies in cows when using the AlphaVision device in diagnosis.

Clinical manifestations	Endometritis	Subinvolution of the uterus	Vaginitis	Cervicitis
Characteristic changes	Cervical canal was slightly dilated, exudate of different nature was noted, in some cases the cervix was enlarged The longitudinal folds of the vaginal part of the cervix were flattened, in some cases there was lumpiness and hyperemia on the surface.	The cervix was flabby without folding, with moderate hyperemia	There were serous-catarrhal deposits on the mucous membrane, swelling and hyperemia, sometimes there were striated or pitting hemorrhages The mucosal folds were smoothed out	The cervix was enlarged, the mucous membrane was flabby, doughy, and sticky; fibrinous deposits, erosions, and ulcers were sometimes found on its surface, and the mucous membrane was enlarged
The nature of the discharge	Catarrhal exudate, in some cases with a mixture of pus	Lochia	Catarrhal exudate, in some cases with a mixture of pus	Purulent-catarrhal or catarrhal exudate

**Table-4:** Clinical picture of obstetric and gynecological pathologies in cows.

Clinical manifestations	Endometritis	Subinvolution of the uterus	Vaginitis	Cervicitis
Common signs of inflammation				
Increase in body temperature, °C	39.5-41.5	39.0-40.5	38.5-39.5	39.0-41.0
Depression and loss of appetite	Present	Present	Absent	Present
The pose of the animal	The animal stands with its back arched, often lying down	The animal stands with its back arched	No changes	The animal stands with its back arched

**Figure-4:**
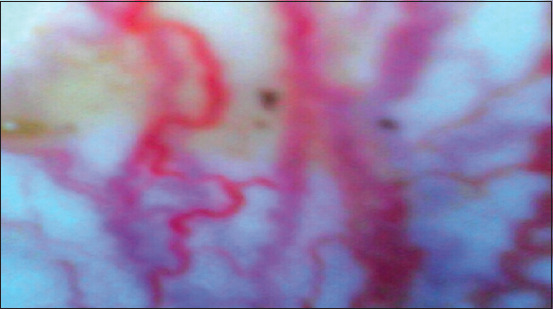
The wall of the vagina with an increased pronounced vascular pattern, the photo is taken using AlphaVision.

Tables-3 and 4 reveal that in the postpartum period in cows with a risk of developing obstetric and gynecological diseases, body temperature can vary depending on the severity of the ongoing changes. When the body temperature changes (increases) in the pattern of the vaginal wall were observed using AlphaVision (Figure-4), it became more pronounced (hemorrhagic). Therefore, observation of the body temperature of animals in the next 24 h is an early sign of the development of pathologies of the reproductive organs. With an increase in body temperature, other physiological indicators, such as respiratory and pulse rates, also increased. The animals refused to feed and drink, lay more, mucopurulent or brownish exudate was released from the birth canal. Rectal palpation of the uterus revealed softness and doughiness, as well as soreness of the uterus; the ovaries (in particular, the right one) were slightly enlarged. According to various criteria, the clinical picture of cervicitis is similar to the symptoms of other diseases of the genital organs, which, in turn, significantly hampered the differentiation of cervicitis as a separate disease, and this, in this case, made the use of the AlphaVision visual insemination system more relevant as it provided a more accurate diagnosis of diseases of the reproductive organs.

### Experiment 3

**Figure-5:**
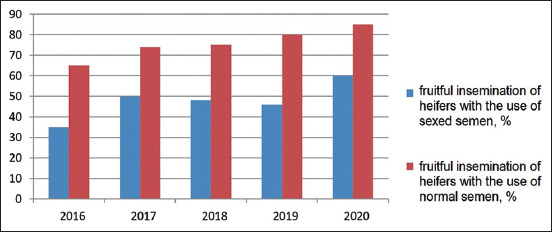
The number of fruitful inseminations, %.

**Figure-6:**
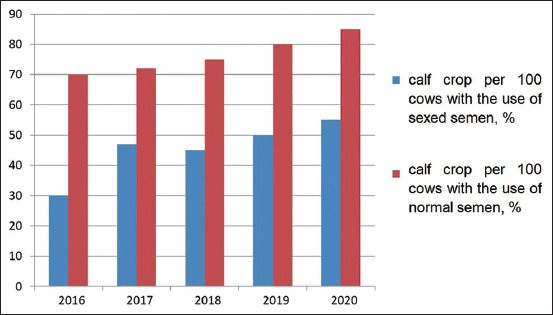
Calf crop per 100 cows, %.

**Figure-7:**
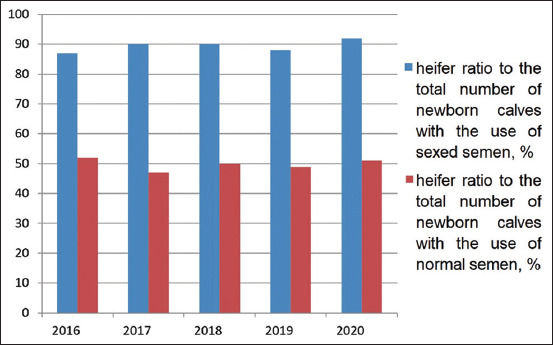
Heifer ratio to the total number of newborn calves, %.

In Turar LLP, Kazakhstan, the calf crop per 100 cows was 70-85% during 2016-2020, with an insemination index of 2.95-2.76 and a service period of 176-154 days, respectively. The study of the influence of sexed semen on the fertility of heifers over the past 5 years (Figure-5) made it possible to conclude that its use reduced the fertility of the animals by an average of 25-30%. The complete data on the use of sexed semen were provided by studies on the number of viable calves obtained, especially heifers (Figures-6 and 7).

The calf crop per 100 cows when using ordinary semen was 70 to 85% throughout the experiment. The same indicator when using sexed semen was 35-50%. Differences in indicators are in the range of 30-40%. Over the entire study period, 253 live calves were obtained from heifers inseminated with sexed semen. The average number of heifers per total number of newborns averaged 85-92% over the years, which is 40% higher than with conventional semen. The percentage of stillborn calves and abortions averaged 5.6%, which does not exceed those figures for the herd as a whole. The insemination index was 2.3 doses.

Studying the data on the effect of sexed semen (Figure-5) on the insemination of heifers, we found a decrease by an average of 30%. In the course of the experiment, we studied the effect of the AlphaVision device (Table-5) on carrying out artificial insemination with sexed semen.

**Table-5:** Fertility of heifers of mating age with the use of the AlphaVision device.

Indicator	AlphaVision Visual Insemination System			

	Control group (semen not divided by sex)		The experimental group (sexed semen)	
	Heads	%	Heads	%
Heifers of mating age	50	100	50	100
Pregnancy at 31-35 day after insemination	46	92	38	76
Actually calved	43	85	30	60

Analyzing the data of our research, it can be noted that the percentage of insemination of heifers during the period from 2016 to 2019 with bisexual semen fluctuated in the range of 65-80%, when AlphaVision was used in 2020 –85% (Table-5), the growth amounted to 5%. The percentage of fruitful insemination in cervical insemination with rectal fixation of the cervix using sexed semen during the period 2016-2019 fluctuated from 35 to 50%. When AlphaVisionin 2020 was used, the figure was 60%, that is, 10% more than with the traditional insemination method.

## Discussion

To increase the level of herd reproduction using innovative devices of insemination (with the AlphaVision device), we conducted a comparative analysis of biotechnological methods of reproduction. The most rational and efficient way of reproduction in the conditions of Northern Kazakhstan was determined. We established the percentage of the first fruitful insemination, 58.3% with cervical insemination with rectal fixation of the cervix and 79.0% by visual insemination using the device AlphaVision. Krymowski [22] showed that only 40% of sperm was placed either in the body of the uterus or evenly distributed between the left and right horns. This study found significant differences between professional technicians and owner technicians. Rather, placement depended on the skill and ability of the individual to determine the position of the rod tip in the reproductive tract. The benefits of using AlphaVision [22] in cattle include the ability to visualize any abnormalities in the vagina or cervix and confirm high fever through the photos taken by observing cervical mucus. The speculum also offers less difficulty in passing the gun through the cervix, as the view of the pharynx prevents the tip from entering the fornix. Physical handling of the cervix is also minimized, which can rule out the potential damage to the lining of the rectum or uterus from excessive harsh handling. These findings are also supported by our study.

One of the disadvantages of using the AlphaVision in cold weather (outdoors) is that cold air enters the vagina through the side slots of the device and can cause vaginal cramping. Of the total number of cows in the herd, 50% of all cows were diagnosed with at least one clinical disease during the 305 days of lactation. Carvalho *el al*. [23] indicated that the aforementioned proportions were similar to those previously reported by researchers in the same study area [14,24,25], who once again emphasized the relevance of postpartum problems in dairy cows.

Moreover, recent studies evaluating data for cattle, namely, diseases of the reproductive organs of cows consistently report long-term effects of uterine infection [24], mastitis [26], and metabolic disturbances [25], and their influence on the milk productivity of dairy cows, so our research focused on diagnosing these diseases.

According to the results of the study on the use of AlphaVision in the growth of the herd with the use of sexed semen, we managed to reach our goals, having obtained results that had scientific and practical significance. It is a well-known fact that high milk production reduces the reproductive qualities of cows. With the introduction of our research into the practical activities of dairy farms in northern Kazakhstan, it will undoubtedly increase the level of the herd reproduction system. Studying the use of sexed semen on the broodstock at Turar LLP, Kazakhstan, we took into account that this technology was very little used in Kazakhstan. We still have no experience in using semen from breeding bulls divided by sex. In its implementation, this technology is very complex; the equipment for the laboratory, in addition to the high cost, requires long-term training of personnel who will operate it. There are no domestic laboratories in the Commonwealth of Independent States that could separate sperm from breeding bulls of local breeds. As a result, the high cost of sexed semen is noted. Like everything new, this technology has not yet taken root very well. However, abroad, this technology is today very popular on family-owned farms, for example, in Britain, where there is a fairly large number of farms engaged in the withdrawal of small livestock (150-300): The ratio of the sale of sexed and ordinary semen from stud bulls is 1:3. In the United States, where there are many large farms, this ratio equals 1:10. Therefore, it was interesting for us to study the use of sexed semen in the northern region of Kazakhstan. We also explored some innovative solutions to reduce the farmer’s costs for the purchase of expensive semen. In our case, the study was carried out on a dairy farm, where the number of lactating cows was more than 1200 heads, and they could afford to purchase sexed semen. During 2016-2019, from 65 to 80% of calves were born from two-sex sperm. In comparison, the percentage of calves with sexed sperm was 35-50%. In the course of the experiment in 2020, we received results of 85-60%, using the sexed semen of 60% of calves. Such fertility after using sorted sperm is consistent with the literature: Tubman *et al*. [27] and Healy *et al*. [28] found rates of 87.8% and 86.0%, respectively. de Jarnette *et al*. [29] and Norman *et al*. [30] reported rates close to the planned 90.0%. These deviations can occur due to different sorting accuracy or due to incomplete or erroneous data recording [28,31].

The sex ratio for normal sperm was different from our assumptions. Most studies obtained between 50% and 52% of male newborns [27,29]. In our study, the ratio changed in this way 49-51% of bulls were born after using conventional sperm, and the number of heifers born from sexed semen was 85-92%. Norman *et al*. [30] also reported 51.5% of calves.

Concerning the productivity of offspring, there are positive studies [28,29] and negative studies [27,29] on the spread of negative consequences for the health of calves obtained from sexed semen. Our study for 2016-2020 showed that the percentage of stillborn calves and abortions averaged 5.6%, which did not exceed these indicators for the herd in this farm as a whole.

## Conclusion

Based on the study carried out, the following conclusions can be drawn.

1. The use of the AlphaVision visual insemination system, as one of the innovative methods of reproduction, made it possible to increase the percentage of fruitful insemination by 20.7%, from 58.3% to 79.0%. The insemination Assist System, equipped with a sealed chamber, has significantly improved the convenience of the technics-inseminator. In particular, it allowed checking the involution of the cow’s cervix, showed pathology, and made it easier to locate it for more painless insemination
2. As a result of our research, we found that with an increase in the age of cows, the incidence of postpartum diseases increased by 30% and that postpartum inflammatory diseases were registered in more than 50% of the surveyed animals. According to various criteria, the clinical picture of cervicitis is similar to the symptoms of other diseases of the genital organs, which, in turn, significantly complicates the differentiation of cervicitis as a separate disease, and this, in this case, makes the use of the AlphaVision visual insemination system more relevant, as it provides a more accurate diagnosis of diseases of the reproductive organs
3. The use of sexed semen reduces the fertilizing capacity of animals by an average of 25-30%. The output of calves per 100 cows when using ordinary semen for the period for 2016-2019 was 65 to 80%, and during the experiment (using AlphaVision) in 2020, it reached 85%, with an increase of 5%. The same indicator when using sexed semen reached 30-50% in 2016-2019, and with AlphaVision in 2020, it showed 60% (a 10% growth). The average number of heifers per total number of newborns, when using sexed semen is on average 85-92%, which is 40% higher than when using conventional semen. The use of sex-segregated semen allows obtaining more heifers from all calves, which, in turn, makes it possible to renew the dairy herd with first-calf heifers in a shorter time and, if necessary, to increase the number of livestock annually
4. Thus, the introduction of the innovative technology described in this work into the practice of farms increases the level of the herd reproduction system.

## Authors’ Contributions

VAR: Conception and design, acquisition and analysis of data, and drafting of the manuscript. AMN: Conception and design and revised the manuscript critically. VAS: Revised the manuscript critically and final approval. AAB: Conception and design of the manuscript. All authors read and approved the final manuscript.
